# BRAF(V600E) mutation together with loss of Trp53 or pTEN drives the origination of hairy cell leukemia from B-lymphocytes

**DOI:** 10.1186/s12943-023-01817-8

**Published:** 2023-08-05

**Authors:** Jiajun Yap, Jimin Yuan, Wan Hwa Ng, Gao Bin Chen, Yuen Rong M. Sim, Kah Chun Goh, Joey Teo, Trixie Y. H. Lim, Shee Min Goay, Jia Hao Jackie Teo, Zhentang Lao, Paula Lam, Kanaga Sabapathy, Jiancheng Hu

**Affiliations:** 1 Division of Cellular and Molecular Research, National Cancer Centre Singapore, 30 Hospital Boulevard, 168583 Singapore, Singapore; 2 Cancer and Stem Cell Program, Duke-NUS Medical School, 8 College Road, 169857 Singapore, Singapore; 3 Department of Urology, The Second Clinical Medical College, The First Affiliated Hospital, Shenzhen People’s Hospital, Jinan University, Southern University of Science and Technology), Shenzhen, 518020 Guangdong China; 4 Geriatric Department, The Second Clinical Medical College, The First Affiliated Hospital, Shenzhen People’s Hospital, Jinan University, Southern University of Science and Technology), Shenzhen, 518020 Guangdong China; 5 Department of Hematology, Singapore General Hospital, Blk7 Outram Road, 169608 Singapore, Singapore; 6 Department of Physiology, National University of Singapore, 2 Medical Drive, 117597 Singapore, Singapore; 7 Cellvec Pte. Ltd, 100 Pasir Panjang Road, 118518 Singapore, Singapore

**Keywords:** Hairy cell leukemia, BRAF(V600E), Tumor suppressor, RAS/RAF/MEK/ERK signaling, B cell biology, B cell lymphoma

## Abstract

Hairy cell leukemia (HCL) is a B-lymphoma induced by BRAF(V600E) mutation. However, introducing BRAF(V600E) in B-lymphocytes fails to induce hematological malignancy, suggesting that BRAF(V600E) needs concurrent mutations to drive HCL ontogeny. To resolve this issue, here we surveyed human HCL genomic sequencing data. Together with previous reports, we speculated that the tumor suppressor TP53, P27, or PTEN restrict the oncogenicity of BRAF(V600E) in B-lymphocytes, and therefore that their loss-of-function facilitates BRAF(V600E)-driven HCL ontogeny. Using genetically modified mouse models, we demonstrate that indeed BRAF(V600E)^KI^ together with Trp53^KO^ or pTEN^KO^ in B-lymphocytes induces chronic lymphoma with pathological features of human HCL. To further understand the cellular programs essential for HCL ontogeny, we profiled the gene expression of leukemic cells isolated from BRAF(V600E)^KI^ and Trp53^KO^ or pTEN^KO^ mice, and found that they had similar but different gene expression signatures that resemble that of M2 or M1 macrophages. In addition, we examined the expression signature of transcription factors/regulators required for germinal center reaction and memory B cell versus plasma cell differentiation in these leukemic cells and found that most transcription factors/regulators essential for these programs were severely inhibited, illustrating why hairy cells are arrested at a transitional stage between activated B cells and memory B cells. Together, our study has uncovered concurrent mutations required for HCL ontogeny, revealed the B cell origin of hairy cells and investigated the molecular basis underlying the unique pathological features of the disease, with important implications for HCL research and treatment.

**Supplementary Information:**

The online version contains supplementary material available at 10.1186/s12943-023-01817-8.

## Key Points

• Hairy cells originate from B-lymphocytes with BRAF(V600E) and TP53 or PTEN mutations.

• Hairy cells exhibit a unique gene expression signature that underlies the pathological features of HCL.

**Supplementary Information:**

The online version contains supplementary material available at 10.1186/s12943-023-01817-8.

## Introduction

HCL is a chronic B cell lymphoma with unique clinicopathological features including pancytopenia, splenomegaly, hepatomegaly, and no lymphadenopathy [1]. Leukemic cells in this disease express a set of specific chemokine receptors and adhesion molecules that direct them to bone marrow, spleen, and hepatic sinusoids rather than lymph nodes, with associated disorders in these organs. The molecular mechanisms that distinguish HCL from other B cell lymphomas and underlie the pathology of this disease remain largely unknown, which impairs the development of precise therapies, particularly for cases that relapse or are refractory to treatment.

Recent genomic studies have identified BRAF(V600E) as a prominent pathogenic driver for HCL, whose reversal extinguishes the distinctive characters of leukemic cells and effectively induces disease regression [2–4]. Introducing BRAF(V600E) in hematopoietic stem cells drives a hematological malignancy with much more aggressive pathology than that of human HCL, which in B cells does not have any lymphoproliferative disorder [5]. Together with previous finding that hairy cell leukemic cells have a gene expression signature resembling that of post-germinal center B cells [6], these studies imply that BRAF(V600E) needs some other concurrent genetic alterations to cooperatively induce hairy cells from B cells [7]. Indeed, cyclin D1 (CCND1) is up-regulated through non-chromosomal translocation in human HCL [8, 9]. The cyclin-dependent kinase inhibitor p27 (CDKN1B) has also been found to be inactivated via genetic mutations or epigenetic silencing [10, 11], which may facilitate leukemic cell clonal expansion. Although it is unclear whether loss of function of TP53 (murine Trp53) contributes to the ontogeny of HCL, TP53 mutations have been found at various frequencies in patient samples [12–15]. Further, p21 (CDKN1A), a key effector of TP53, has been shown inhibited in some HCL cases through mutation of its transcription factor KLF2 or overexpression of its regulatory microRNA [16–18]. These findings suggest that the suppression of pro-apoptotic TP53 signaling may play an essential role in the pathogenesis of HCL. On the other hand, anti-apoptotic PI3K/AKT signaling might be hyperactivated in HCL, likely through overexpression of FLT3 receptor tyrosine kinase as well as activation of the bFGF-FGFR1 autocrine loop [6, 19], or downregulation of PTEN by promoter hypermethylation [20]. In addition, mutations on diverse epigenetic regulators such as MLL3 (KMT2C), KDM6A, CREBBP (CBP), ARID1A and ARID1B have been observed in some HCL patients [14, 16], which may promote the disease progression by turning on oncogenic pathways and off tumor suppressive pathways via epigenetic modifications. How these factors contribute to the BRAF(V600E)-driven origin of hairy cells and disease progression has not been determined yet.

In this study, we combined BRAF(V600E) knockin with various tumor suppressor knockouts in B cells to examine the origin and pathology of HCL, and hence constructed animal models mimicking human disease.

## Results

### BRAF(V600E)^KI^ together with Trp53^KO^ or pTEN^KO^ but not P27^KO^ induces lymphoproliferative disorder in murine B-lymphocytes

**Fig. 1:**
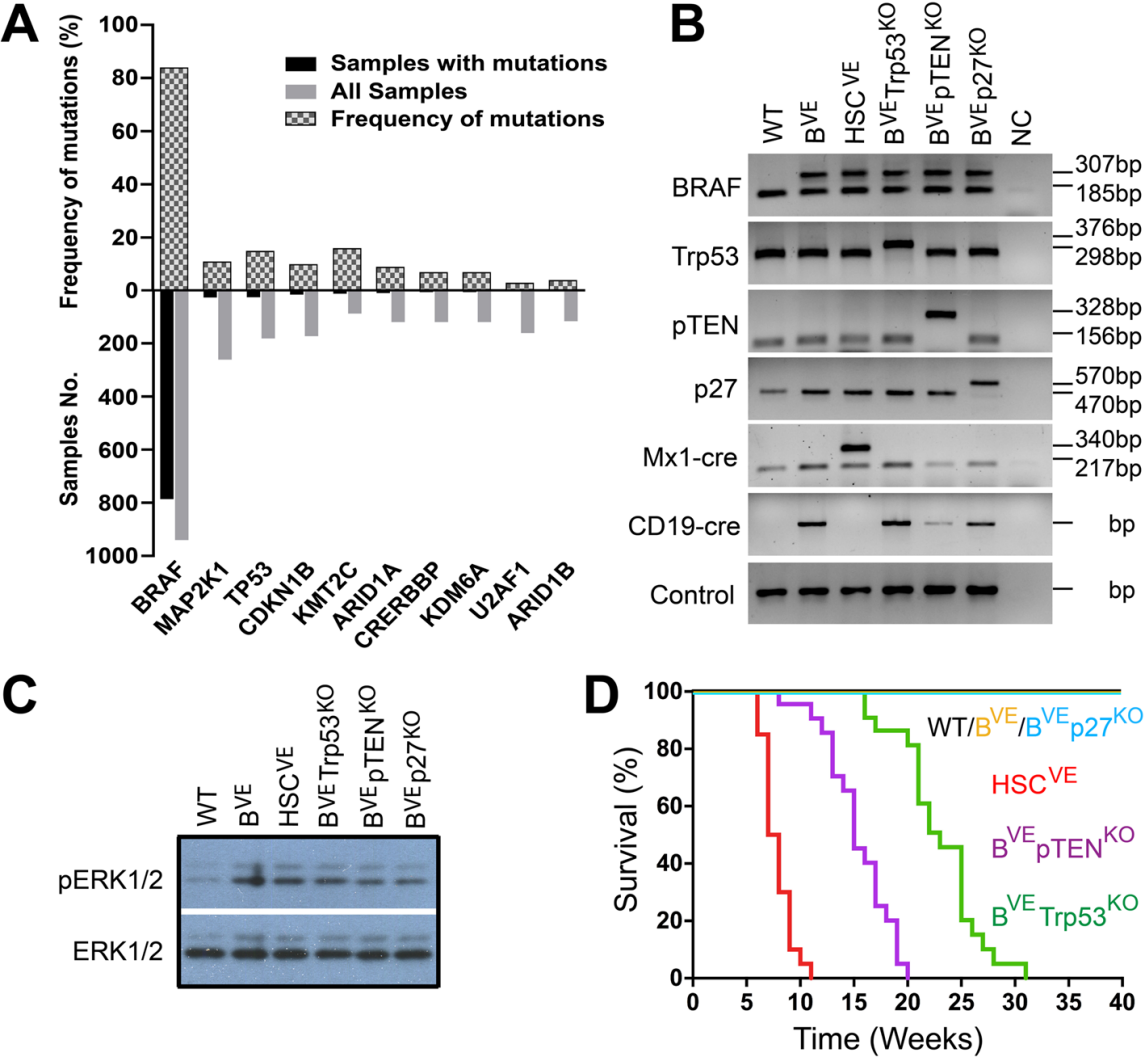
Loss of Trp53 or pTEN but not P27 facilitates the development of BRAF(V600E)-driven B cell malignancies. **A** Statistical analysis of genetic mutations in human HCL. The data shows human HCL cases extracted from the COSMIC database and those cases reported in the literatures. The frequencies of high-prevalent gene mutations were calculated and were plotted. **B** Genetic modifications in mice models. Genomic DNA was isolated from tails of 3-week-old wild-type and mutant mice, and genetic modifications were detected with PCR and agarose gel analysis. **C** The expression of BRAF(V600E) in B-lymphocytes activates ERK signaling. Splenic B cells were isolated from 5-week-old wild-type or mutant mice by using anti-CD19 MACS and lysed for anti-phospho-ERK1/2 immunoblot. **D** A Kaplan-Meier plot of mice life span. The overall survival of mice was followed up to 40 weeks (*n* = 20). All images are representative of at least three independent experiments

To identify concurrent genetic alterations that facilitate the BRAF(V600E)-driven origin of hairy cells, we firstly extracted genomic sequencing data of HCL cases from the COSMIC (Catalogue of Somatic Mutations in Cancer) database, the ICGC (International Cancer Genome Consortium) database, and the cBioportal for Cancer Genomics database, and examined all pronounced mutations. As shown in Fig. 1A and Table S1, ~ 85% of HCL patient samples (including HCL variant) carried a BRAF(V600E) mutation, while ~ 10% of cases harbored constitutively active MAP2K1 (MEK1) mutations, suggesting that hyperactive ERK signaling plays a determinant role in the ontogeny of HCL. In contrast to highly prevalent mutations in oncogenic ERK signaling, tumor suppressor mutations were much less frequent, with ~ 15% of samples harboring TP53 mutations and ~ 10% P27 (CDKN1B) mutations. In addition, low-frequency mutations were identified on diverse epigenetic regulators including MLL3 (KMT2C, ~ 16%), ARID1A (~ 9%), CRERBBP (CBP, ~ 7%), KDM6A (~ 7%), and ARID1B (~ 4%), suggesting that epigenetic modifications may contribute to the pathogenesis of HCL. Overall, this data is consistent with previous reports [14, 16], and supports the notion that BRAF(V600E) cooperates with other alterations to drive the ontogeny of HCL. Given the loss-of-function of TP53 and P27 as well as hyperactivation of PI3K/AKT signaling in HCL [6, 21], we next determined whether BRAF(V600E) mutation together with a knockout of Trp53, p27 or pTEN in mice induced a leukemic syndrome resembling human HCL. Hence, we bred BRAF^CA^ mice with Trp53^flox/flox^ or p27^flox/flox^ or pTEN^flox/flox^ mice as well as cd19^cre^ mice, and obtained BRAF^CA/+^Trp53^flox/flox^cd19^cre/+^, BRAF^CA/+^p27^flox/flox^cd19^cre/+^, BRAF^CA/+^pTEN^flox/flox^cd19^cre/+^ and BRAF^CA/+^cd19^cre/+^ strains, which are henceforth referred as B^VE^P53^−/−^, B^VE^P27^−/−^, B^VE^PTEN^−/−^, and B^VE^ strains respectively. In contrast to the BRAF^CA/+^Mx1^cre/+^ strain that turns on BRAF(V600E) in hematopoietic stem cells (henceforth referred as HSC^VE^ strain), these strains constitutively expressed BRAF(V600E) that activates ERK signaling with loss of individual tumor suppressors only in B cells, as confirmed by PCR and immunoblots against phospo-ERK1/2, phospho-AKT, Trp53 and P27 in B cell lysates (Fig. 1B, C and S1). As reported before, HSC^VE^ mice developed a very aggressive hematopoietic malignancy and died at ~ 8 weeks, while B^VE^ mice did not exhibit any proliferative disorders and had a normal lifespan (Fig. 1D), suggesting that BRAF(V600E)-driven cellular malignancy requires an appropriate landscape. Unlike HSC^VE^ mice, B^VE^P53^−/−^ and B^VE^PTEN^−/−^ mice developed less aggressive malignant disorders and died at ~ 24 weeks or ~ 16 weeks respectively, while B^VE^P27^−/−^ mice did not experience any malignant syndromes and had a normal lifespan as B^VE^ mice (Fig. 1D). This data indicates that loss-of-function of TP53 or PTEN but not P27 facilitates BRAF(V600E)-induced B cell malignancy.

### BRAF(V600E)^KI^ together with Trp53^KO^ or pTEN^KO^ in murine B-lymphocytes drives a chronic B cell lymphoma with blood parameters resembling that of human HCL

**Fig. 2:**
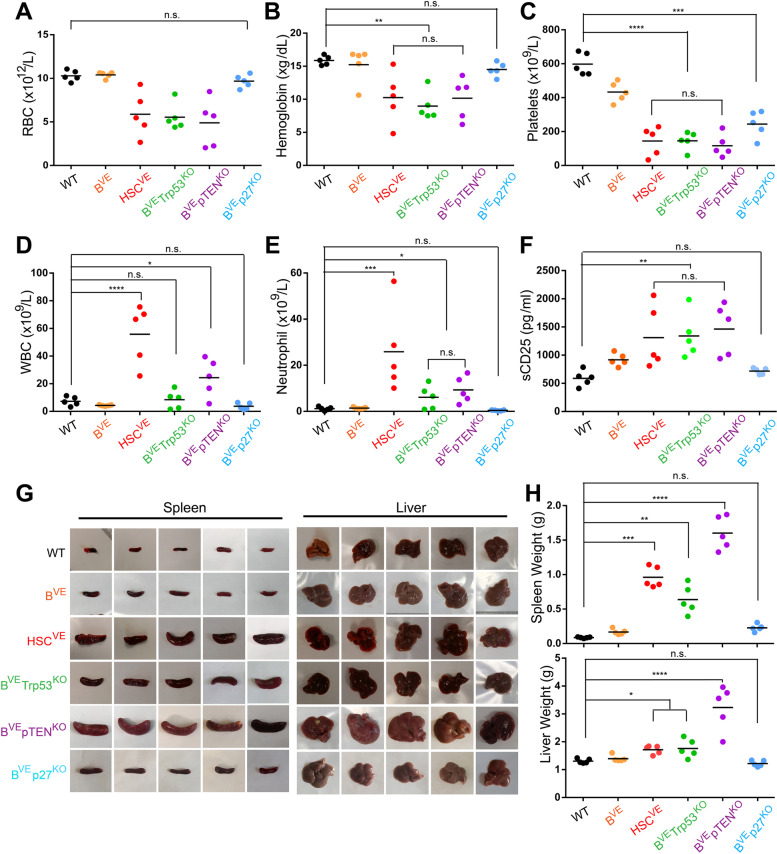
B^VE^Trp53^KO^ or B^VE^pTEN^KO^ but not B^VE^P27^KO^mice develop hematopoietic disorders with splenomegaly and hepatomegaly like HSC^VE^ mice. **A**-**E** Blood parameters were altered in HSC^VE^, B^VE^P53^−/−^, and B^VE^PTEN^−/−^ mice but not B^VE^P27^−/−^ mice. Blood was collected from the tail veins of mice with terminal disease (HSC^VE^, 8 ~ 12 weeks; B^VE^P53^−/−^, 25 ~ 30 weeks; and B^VE^PTEN^−/−^, 16 ~ 20 weeks) and blood parameters were measured with a hematology analyzer. A, Red blood cell counts; B, hemoglobin counts; C, platelet counts; D, white blood cell counts; and E, neutrophil counts of mice at the late stage of disease (*n* = 5, *p < 0.05, **p < 0.01, ***p < 0.001, ****p < 0.0001; n.s., not significant). **F** High level of sCD25 was indicated in HSC^VE^, B^VE^P53^−/−^, and B^VE^PTEN^−/−^ mice but not in B^VE^P27^−/−^ mice. The serum concentration of sCD25 in mice with late disease stage was measured by ELISA (*n* = 5, **p < 0.01; n.s., not significant). **G**-**H** HSC^VE^, B^VE^P53^−/−^, and B^VE^PTEN^−/−^ mice developed splenomegaly and hepatomegaly. The spleens and livers of mice with terminal stage disease were harvested (**G**) and weighed (**H**) at the experimental end point. In all experiments, 28-week-old wild type or B^VE^ or B^VE^P27^−/−^ mice served as controls (*n* = 5, *p < 0.05, **p < 0.01, ***p < 0.001, ****p < 0.0001; n.s., not significant)

Since B^VE^P53^−/−^ and B^VE^PTEN^−/−^ mice developed chronic hematopoietic malignancies, we next determined whether these malignancies possessed the pathological features of human HCL. We first measured the blood parameters of B^VE^P53^−/−^ and B^VE^PTEN^−/−^ mice at the terminal stage of disease. As shown in Fig. 2A, B, red blood cell counts and blood hemoglobin content in B^VE^P53^−/−^ and B^VE^PTEN^−/−^ mice was dramatically decreased like HSC^VE^ mice, while B^VE^ and B^VE^P27^−/−^ mice had no significant change, suggesting impaired bone marrow hematopoiesis in B^VE^P53^−/−^ and B^VE^PTEN^−/−^ mice. Consistently, the peripheral blood platelets also decreased strikingly in these two mice strains like in HSC^VE^ mice, whereas these alterations were much less pronounced in B^VE^ and B^VE^P27^−/−^ mice strains (Fig. 2C). However, unlike HSC^VE^ mice, B^VE^P53^−/−^ and B^VE^PTEN^−/−^ mice showed no change or slightly up-regulate their white blood cell and neutrophil counts in peripheral blood, akin to human HCL (Fig. 2D, E), though all three strains had a high level of serum CD25 (Fig. 2F). Altogether, B^VE^P53^−/−^ and B^VE^PTEN^−/−^ mice had blood pathological features resembling that of human HCL.

### BRAF(V600E)^KI^ together with Trp53^KO^ or pTEN^KO^ in murine B-lymphocytes induces splenomegaly and hepatomegaly

Leukemic cells home to bone marrow and disrupt hematopoiesis in human HCL, which results in pancytopenia. B^VE^P53^−/−^ and B^VE^PTEN^−/−^ mice exhibited this pathological feature, which led us to determine whether these mice have other pathological features of human HCL such as splenomegaly and hepatomegaly and serve as an animal model of human disease. Therefore we harvested spleens and livers from B^VE^P53^−/−^ and B^VE^PTEN^−/−^ mice at the terminal stage of disease, and found that both mice strains had enlarged spleens and livers (Fig. 2G-H) same as HSC^VE^ mice, and in contrast to wild-type, B^VE^ and B^VE^P27^−/−^ mice. Particularly, B^VE^PTEN^−/−^ mice had more aggressive splenomegaly and hepatomegaly phenotypes than B^VE^P53^−/−^ mice, resembling a more aggressive subtype of human HCL with high PI3K/AKT signaling and a poor prognosis [21].

### B lymphocyte-specific BRAF(V600E)^KI^ together with Trp53^KO^ or pTEN^KO^ induces a malignant syndrome distinct from that triggered by HSC-specific BRAF(V600E)^KI^

**Fig. 3:**
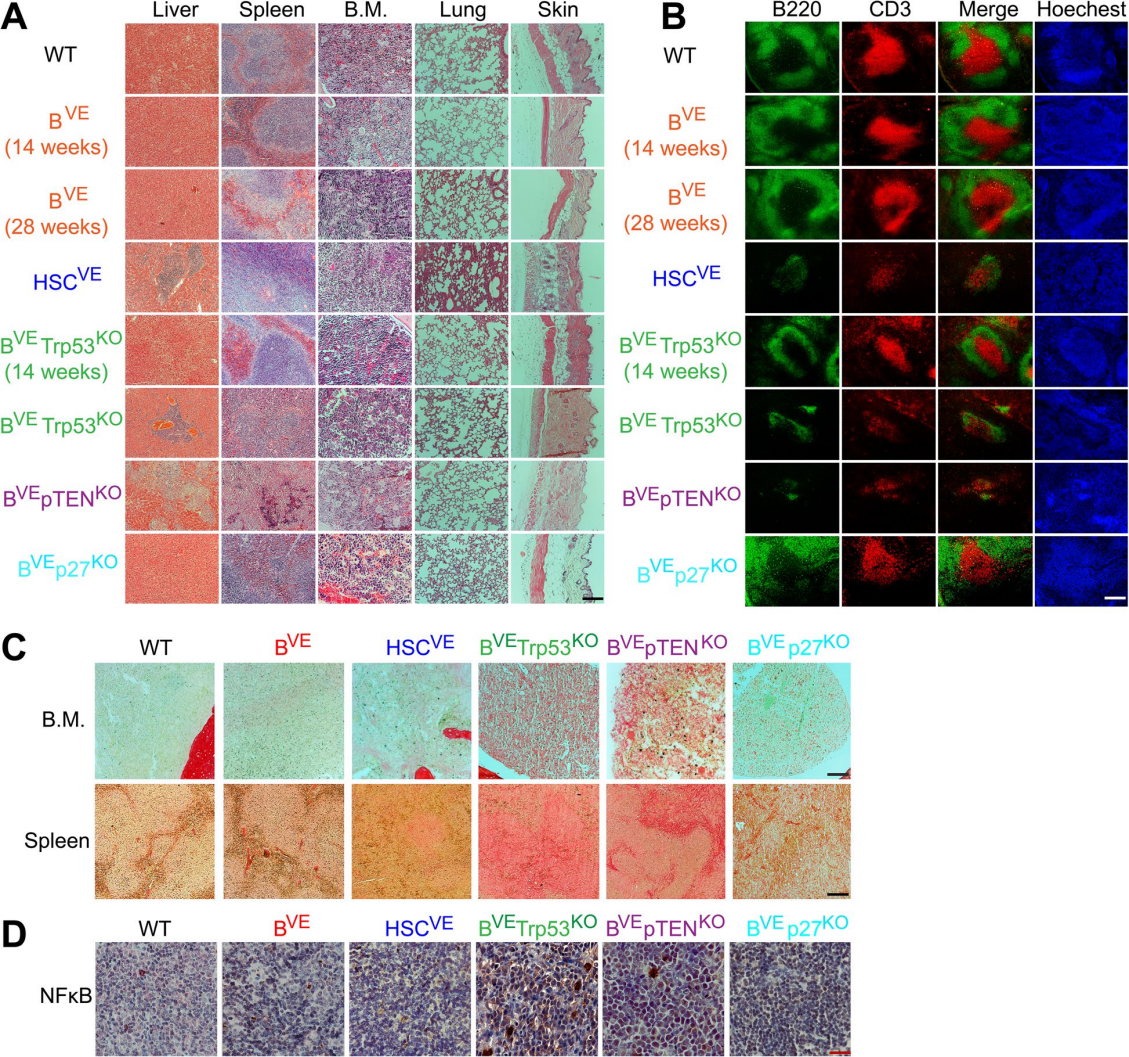
Unique pathological features of malignancies in B^VE^Trp53^KO^ or B^VE^pTEN^KO^ mice. **A** Pathological alterations in tissues or organs of HSC^VE^, B^VE^Trp53^KO^ or B^VE^pTEN^KO^ mice. Histological analysis of tissues or organs was carried out as described in Materials and Methods. Scale = 100 μm. **B** The disruption of splenic architecture in HSC^VE^, B^VE^P53^−/−^ and B^VE^PTEN^−/−^ mice. Frozen spleen sections were stained with anti-B220 AF488 (green) for B cell zones, anti-CD3 AF549 (red) for T cell zones, and Hoechst for cellular nuclei as stated in Materials and Methods. Scale = 100 μm. **C** Fibrosis of bone marrow and spleen was induced by leukemic cells strongly in B^VE^P53^−/−^ and B^VE^PTEN^−/−^ mice, but weakly in HSC^VE^ mice. The bone marrow and spleen sections were stained with Picro Sirius Red for collagen. Scale = 100 μm. **D** NFκB signaling was activated in the spleen of B^VE^P53^−/−^ mice but not other mice. The spleen sections were stained with anti-NFκB (active units) antibody as in Materials and Methods, and the positive signal was shown as brown color. Scale = 50 μm. All tissues were harvested from mice with disease at terminal stage or mice without disease at 28 weeks or indicated age. All images are representative of at least five mice per group and three independent experiments

Although HSC^VE^, B^VE^P53^−/−^ and B^VE^PTEN^−/−^ mice developed a malignant syndrome with pancytopenia, splenomegaly and hepatomegaly resembling human HCL, the pathology of HSC^VE^ mice was much more aggressive than in B^VE^P53^−/−^ and B^VE^PTEN^−/−^ mice. To understand underlying mechanisms, we extracted various organs/tissues from these mice and examined potential pathological alterations by histological staining. As shown in Fig. 3A, B^VE^P53^−/−^ and B^VE^PTEN^−/−^ mice had apparent pathological changes in liver, spleen, and bone marrow like HSC^VE^ mice. In liver, there were typical leukemic cell infiltrates, particularly angiomatous lesions with red blood cell lakes in B^VE^P53^−/−^ mice. In the spleen, the red pulp was diffusely infiltrated by mononuclear cells and the white pulp showed apparent atrophy, which disrupts splenic anatomical structure. In bone marrow, most leukemic cells of HSC^VE^ mice exhibited normal morphology except slightly larger cytoplasm, while leukemic cells of B^VE^P53^−/−^ and B^VE^PTEN^−/−^ mice had heterogeneous morphology with some giant leukemic cells having lobulated nuclei. In addition, HSC^VE^ mice also showed striking pathogenic alterations in lung and skin. This mouse strain developed severe pulmonary histiocytosis as well as skin inflammation (Fig. 3A right two columns and S3). Together, these data indicate that BRAF(V600E) mutations in hematopoietic stem cells and B lymphocytes induce quite different malignancies.

To further characterize the malignancies in HSC^VE^, B^VE^P53^−/−^ and B^VE^PTEN^−/−^ mice, we next comprehensively evaluated the pathological alterations in their blood, bone marrow and spleen by using immunological and immunohistochemistry methods. We extracted immune cells from these tissues/organs and stained with immunofluorescent antibodies. Using flow cytometry, we found that lymphocyte components were dramatically altered in these tissues/organs from HSC^VE^, B^VE^P53^−/−^ and B^VE^PTEN^−/−^ mice. Briefly, all strains had less T cells and B cells as well as a reversal of the B/T cell ratio in blood and spleen (Figure S3A, B), and less total B cells but a higher mature B cell (IgD^+^) ratio in bone marrow (Figure S3C). Further, we examined by immunohistochemistry staining potential structural alterations on splenic functional compartments in HSC^VE^, B^VE^P53^−/−^ and B^VE^PTEN^−/−^ mice. As shown in Fig. 3B, both T cell and B cell staining was very weak and there were no visible T cell zones and B cell zones in spleens from HSC^VE^, B^VE^P53^−/−^ and B^VE^PTEN^−/−^ mice, consistent with our finding from flow cytometry analysis that splenic T cells and B cells were extremely reduced in number in these mice. Since human hairy leukemic cells home to bone marrow and spleen and induce the fibrosis of these organs, we determined whether this occurred in HSC^VE^, B^VE^P53^−/−^ and B^VE^PTEN^−/−^ mice through anti-collagen immunohistochemistry staining, and found indeed that there were variable fibrosis in both bone marrow and spleen from these mice strains, with B^VE^P53^−/−^ ≈ B^VE^PTEN^−/−^ > HSC^VE^ (Fig. 3C). Moreover, we also checked the NFκB signal in spleen sections from these mice strains, given its potential role in spleen fibrosis of human HCL [22]. However, only the spleen section from B^VE^P53^−/−^ mice exhibited positive staining for phospho-NFκB (Fig. 3D), suggesting that leukemic cell infiltration-induced fibrosis may be achieved through activating different signaling pathways in HCL.

### Leukemic cells from HSC^VE^, B^VE^P53^−/−^ and B^VE^PTEN^−/−^ mice have distinct cellular features

**Fig. 4:**
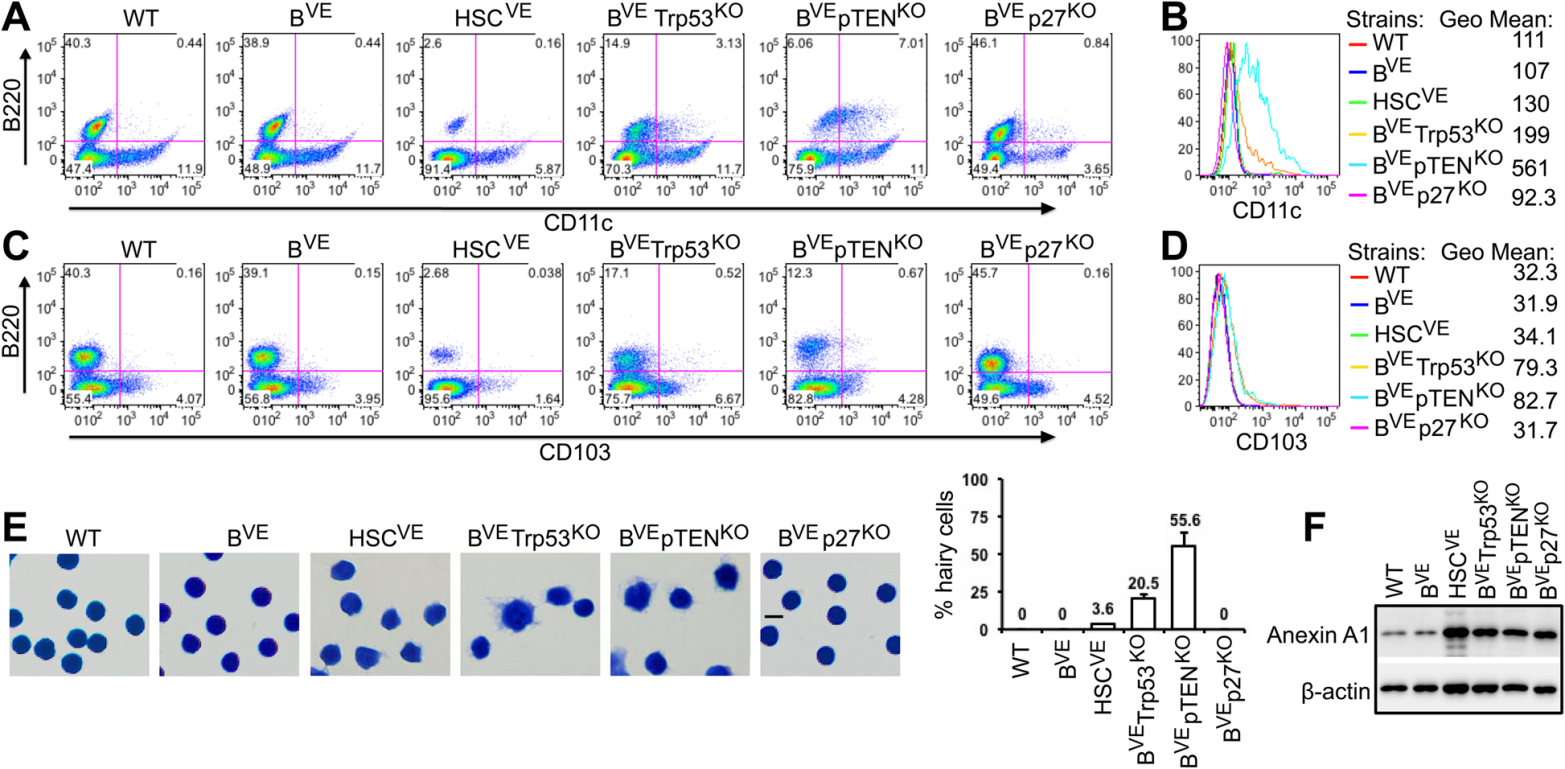
Splenic B^VE^Trp53^KO^ or B^VE^pTEN^KO^ leukemic cells resemble hairy cells in human HCL. **A**-**D** Differential expression of human HCL markers (CD11c and CD103) by splenic B cells of HSC^VE^, B^VE^P53^−/−^, and B^VE^PTEN^−/−^ mice at the terminal stage of disease. Splenocytes were stained with anti-B220 and CD11c (A-B) or CD103 (C-D) antibodies and analyzed by flow cytometry. A and C show 2D plots of B220 versus CD11C or CD103. B and D show histograms and geometric means of CD11c or CD103 in B220^+^ populations. **E** The HSC^VE^, B^VE^P53^−/−^, and B^VE^PTEN^−/−^ leukemic cells exhibited different cellular morphologies from normal B-lymphocytes. Splenic B cells isolated from HSC^VE^, B^VE^P53^−/−^, and B^VE^PTEN^−/−^ mice at the terminal stage of disease were stained with Giemsa and imaged by a microscope. Hairy projections were observed on most B^VE^PTEN^−/−^ leukemic cells, partially on B^VE^P53^−/−^ leukemic cells, and rarely on HSC^VE^ leukemic cells (Scale = 10 μm). The ratios of hairy cells in purified leukemic cells were calculated manually and represent average values from at least five samples. **F** HSC^VE^, B^VE^P53^−/−^, and B^VE^PTEN^−/−^ leukemic cells highly expressed the human HCL marker, Annexin A1. Whole lysates of splenic B cells isolated from different mice strains with or without disease was measured for the expression of Annexin A1 by SDS-PAGE and immunoblot. In all experiments, splenocytes or B cells isolated from 28-week-old wild type or B^VE^ or B^VE^P27^−/−^ mice served as controls. All images are representative of at least five mice per group and three independent experiments

Since B^VE^P53^−/−^ and B^VE^PTEN^−/−^ mice developed a syndrome resembling human HCL, we next investigated whether leukemic cells in these two mice strains had the cellular phenotype of hairy cells in human disease. Firstly, we checked by immunofluorescent staining the expression of two typical hairy cell markers, CD11c and CD103 on splenic leukemic cells from HSC^VE^, B^VE^P53^−/−^ and B^VE^PTEN^−/−^ mice that have developed the syndrome. As shown in Fig. 4A, B and S4A, the whole population of B220^+^ splenic B cells from B^VE^PTEN^−/−^ mice significantly up-regulated expression of CD11c, and B cells from B^VE^P53^−/−^ mice exhibited a heterogeneous expression of CD11c (~ 25% CD11c positive cells), while B cells from HSC^VE^ mice had little-to-no expression of CD11c. As for CD103, only a tiny population of splenic B cells from B^VE^P53^−/−^ and B^VE^PTEN^−/−^ mice slightly up-regulated the expression of this marker (Fig. 4C, D and S4B). Secondly, we purified the splenic B cells from HSC^VE^, B^VE^P53^−/−^ and B^VE^PTEN^−/−^ mice strains with malignant syndrome (Figure S5) and carried out a morphological analysis. Like human hairy cells, a significant population of splenic B cells from B^VE^P53^−/−^ and B^VE^PTEN^−/−^ mice strains at the terminal stage of disease exhibited an enlarged cell body with projections, a typical morphology of human hairy cells. In contrast, splenic B cells from HSC^VE^ mice rarely had projections on the cell surface from an enlarged cell body (Fig. 4E). In addition, we found by immunoblot that splenic B cells from HSC^VE^, B^VE^P53^−/−^, B^VE^PTEN^−/−^ or B^VE^P27^−/−^ mice expressed high levels of Annexin A1, a typical marker for human HCL (Fig. 4F). Together, these data indicated that both B^VE^P53^−/−^ and B^VE^PTEN^−/−^ mice develop a bona fide HCL syndrome and the latter may represent for an aggressive subtype with poor prognosis [21].

### Leukemic cells from HSC^VE^, B^VE^P53^−/−^ and B^VE^PTEN^−/−^ mice have distinct gene expression signatures

**Fig. 5:**
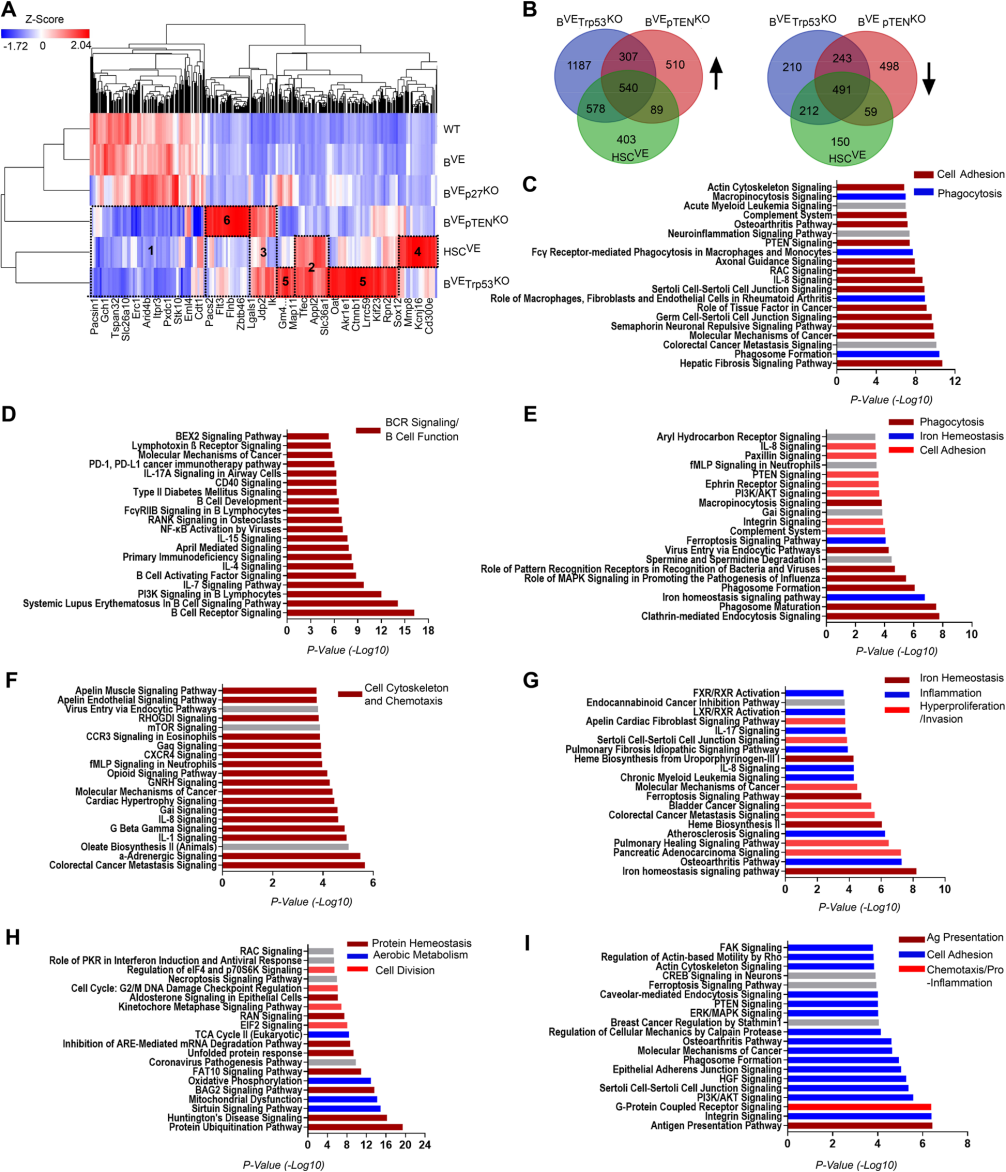
Splenic B^VE^Trp53^KO^ or B^VE^pTEN^KO^ leukemic cells have unique gene expression signatures. **A** The lineage-specific gene expression signatures in HSC^VE^, B^VE^P53^−/−^ or B^VE^PTEN^−/−^ leukemic cells were explored by RNA-seq analysis. RNA samples (three samples per group) were extracted from purified splenic B cells and were analyzed by next-generation sequencing. The hierarchical clustering heatmap was generated as described in Materials and Methods. Signature #1, gene clusters down-regulated in all three leukemic cells; Signature #2, gene clusters up-regulated in HSC^VE^ and B^VE^P53^−/−^ leukemic cells; Signature #3 gene clusters up-regulated in B^VE^P53^−/−^ and B^VE^PTEN^−/−^ leukemic cells; Signature #4, #5, and #6, lineage-specific gene clusters up-regulated in HSC^VE^, B^VE^P53^−/−^ or B^VE^PTEN^−/−^ leukemic cells. **B** A Venn diagram of genes that were up-regulated (upper panel) or down-regulated (lower panel) in HSC^VE^, B^VE^P53^−/−^ and B^VE^PTEN^−/−^ leukemic cells. **C**-**I** Differential cellular programs were turned on in HSC^VE^, B^VE^P53^−/−^ and B^VE^PTEN^−/−^ leukemic cells. Ingenuity pathway analyses (IPA) of prominent gene signatures of HSC^VE^, B^VE^P53^−/−^ or B^VE^PTEN^−/−^ leukemic cells shown in the hierarchical heatmap (**A**) or the Venn diagrams (**B**) was calculated as described in the Materials and Methods, and then the pathways that regulate approximate cellular function were categorized accordingly. C-D, Cellular activities were enhanced (**C**) or dampened (**D**) in all three leukemic cells. E-F, Cellular activities were up-regulated in HSC^VE^ and B^VE^P53^−/−^ leukemic cells (**E**) or B^VE^P53^−/−^ and B^VE^PTEN^−/−^ leukemic cells (**F**). G-I, Cellular activities were elevated in HSC^VE^ leukemic cells (**G**), or B^VE^P53^−/−^ leukemic cells (**H**), or B^VE^PTEN^−/−^ leukemic cells (I). In all experiments, splenic B cells were isolated from mice with terminal stage disease or mice without disease at 28 weeks. All data are representative of three mice per group

HSC^VE^, B^VE^P53^−/−^ and B^VE^PTEN^−/−^ mice developed similar but different malignant syndromes, suggesting that BRAF(V600E) drives distinct cellular programs in different genetic landscapes (hematopoietic stem cells versus B lymphocytes and P53^KO^ versus pTEN^KO^). To understand cellular programs driven by BRAF(V600E) for HCL pathogenesis, we extracted mRNAs from splenic leukemic cells in HSC^VE^, B^VE^P53^−/−^ and B^VE^PTEN^−/−^ mice and determined gene expression profiles by RNA-seq. As shown in Fig. 5A and B, splenic leukemic cells in these mice strains had quite different gene expression patterns though having significant co-regulated genes. We next carried out functional analyses of prominent gene clusters that changed in these leukemic cells by using the Ingenuity Pathway Analysis (IPA) platform. Leukemic cells from all three malignant strains expressed genes involved in cellular adhesion and phagocytosis at high levels (Fig. 5C), and down-regulated those essential for antigen receptor signaling and function of B lymphocytes (Fig. 5D). This suggests that these leukemic cells acquired a feature of invasive and phagocytic cells and lost their original function as B lymphocytes. Besides these gene clusters that overlapped in all leukemic cells, B^VE^P53^−/−^ leukemic cells had two up-regulated gene clusters shared with either HSC^VE^ leukemic cells or B^VE^PTEN^−/−^ leukemic cells respectively. These clusters facilitate phagocytosis, iron homeostasis, and adhesion of HSC^VE^ and B^VE^P53^−/−^ leukemic cells (Fig. 5E), or cytoskeletal rearrangement and chemotaxis of B^VE^P53^−/−^ and B^VE^PTEN^−/−^ leukemic cells (Fig. 5F). More importantly, all leukemic cells had their own lineage-specific up-regulated gene clusters that contribute to iron hemostasis, inflammation & hyperproliferation, and invasion in HSC^VE^ leukemic cells (Fig. 5G), or protein hemostasis and aerobic metabolism, and cell division in B^VE^P53^−/−^ leukemic cells (Fig. 5H), or cellular adhesion, antigen presentation, and pro-inflammatory activity in B^VE^PTEN^−/−^ leukemic cells (Fig. 5I). In summary, although all leukemic cells have some common features, HSC^VE^ leukemic cells resemble acute leukemic cells that have higher levels of iron metabolism, cellular proliferation and invasion, whereas B^VE^P53^−/−^ leukemic cells are close to M2 macrophages by virtue of enhanced aerobic metabolism, biomaterial turnover and phagocytosis, and B^VE^PTEN^−/−^ leukemic cells are similar to M1 macrophages with much stronger pro-inflammatory signaling, chemotaxis, and antigen presentation ability.

**Fig. 6:**
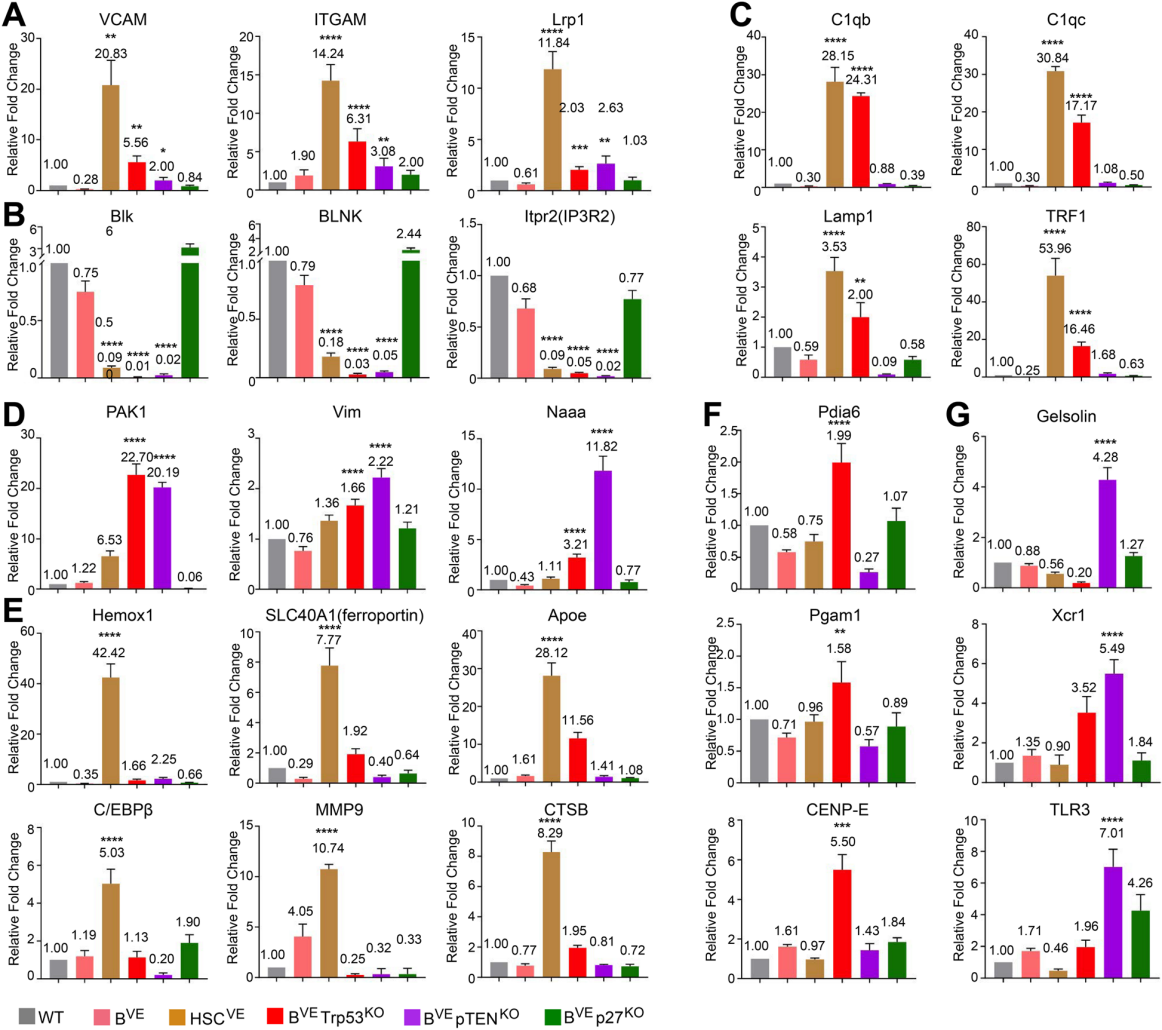
Quantitative PCR analysis of representative genes expressed by splenic HSC^VE^, B^VE^Trp53^KO ^or B^VE^pTEN^KO ^leukemic cells. **A**-**G** Quantitative PCR validation of representative genes in cellular programs altered in HSC^VE^, B^VE^P53^−/−^ or B^VE^PTEN^−/−^ leukemic cells. The qPCR analysis of representative genes was carried out as in Materials and Methods. A, representative genes for cell adhesion and phagocytosis that are up-regulated in all three leukemic cells. B, representative genes for BCR signaling or B cell function that are down-regulated in all three leukemic cells. C, representative genes for phagocytosis, iron homeostasis, and cell adhesion up-regulated in HSC^VE^ and B^VE^P53^−/−^ leukemic cells. D, representative genes for cell cytoskeleton and chemotaxis that are up-regulated in both B^VE^P53^−/−^ and B^VE^PTEN^−/−^ leukemic cells. E, representative genes for cellular iron homeostasis, inflammation and hyperproliferation/invasion that are highly expressed by HSC^VE^ leukemic cells. F, representative genes for protein homeostasis and aerobic metabolism that are highly expressed by B^VE^P53^−/−^ leukemic cells. G, representative genes for cell adhesion, antigen presentation, and pro-inflammatory signaling that are highly expressed by B^VE^PTEN^−/−^ leukemic cells. In all experiments, splenic B cell RNAs were isolated from mice with disease at terminal stage or mice without disease at 28 weeks. All data are representative of at least five mice per group and three independent experiments. *p < 0.05, **p < 0.01, ***p < 0.001, ****p < 0.0001

To confirm our RNA-seq data and bioinformatics analysis, we carried out quantitative PCR validation of representative genes in different clusters. VCAM, ITGAM and LRP1 that have an important role in cell adhesion or phagocytosis [23–25] were significantly up-regulated in all leukemic cells, whilst key factors for antigen receptor signaling of B lymphocytes such as Blk, BLNK and Itpr2 (IP3R2) [26] were severely dampened (Fig. 6A, B). Consistently, C1qb, C1qc, Lamp1 and Trf1 (transferrin receptor 1) that facilitate cellular phagocytosis or iron homeostasis [26–29] were also elevated in HSC^VE^ and B^VE^P53^−/−^ but not B^VE^PTEN^−/−^ leukemic cells (Fig. 6C). PAK1, Vimentin, and Naaa that mediate cellular cytoskeletal rearrangement and chemotaxis [29–32] were remarkably up-regulated in B^VE^P53^−/−^ and B^VE^PTEN^−/−^ but not HSC^VE^ leukemic cells (Fig. 6D). As for the lineage-specific expressed genes, Hemox1, SLC40A1 (ferroportin), ApoE, C/EBPβ, MMP9 and Ctsb that enhance iron storage, cellular proliferation and invasion [32–38] were highly expressed in HSC^VE^ leukemic cells (Fig. 6E), whereas Pdia6, Pgam1 and CENP-E that are key enzymes/regulators for protein turnover, aerobic metabolism, or cell division [38–41] were highly up-regulated in B^VE^P53^−/−^ leukemic cells (Fig. 6F) In contrast, Gelsolin, Xcr1, TLR3 that strengthen cell adhesion, antigen presentation or proinflammatory signaling [41–44] were significantly increased in B^VE^PTEN^−/−^ leukemic cells (Fig. 6G). Overall, these data support our conclusion that HSC^VE^ leukemic cells exhibit a gene expression pattern resembling acute leukemia while B^VE^P53^−/−^ and B^VE^PTEN^−/−^ leukemic cells have a transcriptional phenotype close to that of M2 or M1 macrophages respectively.

### Leukemic cells from B^VE^P53^−/−^ and B^VE^PTEN^−/−^ strains down-regulate transcription factors for germinal center reaction and differentiation of activated B cells

**Fig. 7:**
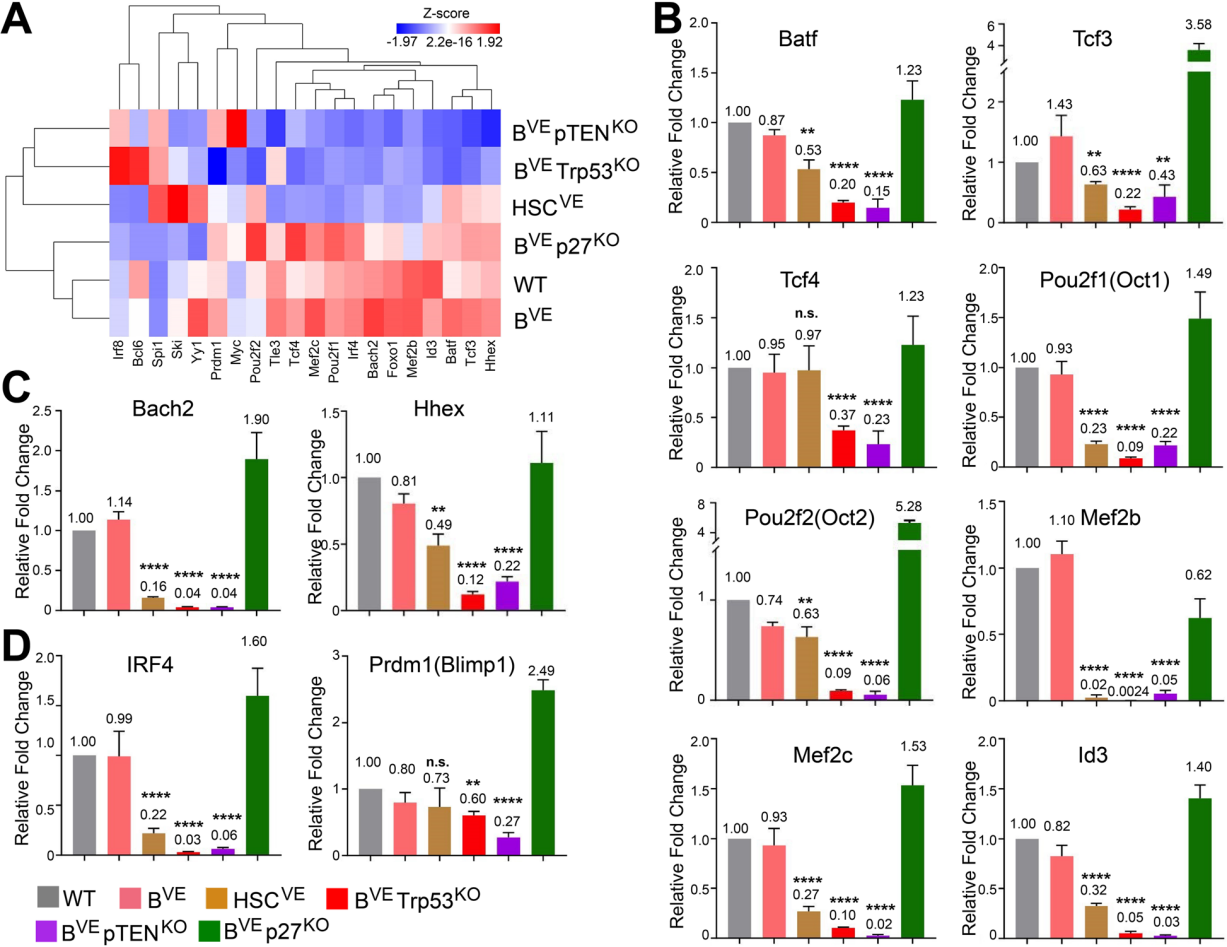
Transcription factors and regulators of germinal center reaction and memory B cells versus plasma cells differentiation are severely suppressed in splenic B^VE^Trp53^KO^ or B^VE^pTEN^KO^ leukemic cells. **A** Expression of transcription factors/regulators that are involved in germinal center reaction and lineage differentiation of memory B cells versus plasma cells was altered in HSC^VE^, B^VE^P53^−/−^, and B^VE^PTEN^−/−^ leukemic cells. Expression data for 20 transcription factors/regulators was extracted from the whole RNA-seq data sets of splenic B cells or leukemic cells from wild type, B^VE^, HSC^VE^, B^VE^P53^−/−^, B^VE^PTEN^−/−^ and B^VE^P27^−/−^ mice, and a hierarchical clustering heatmap was generated as in Materials and Methods. **B**-**D** Quantitative PCR validation of expression of transcription factors/regulators for germinal center reaction (**B**), memory B cell commitment (**C**), and plasma cell commitment (**D**). In all experiments, splenic B cell RNAs were isolated from mice with terminal stage disease or mice without disease at 28 weeks. All data in B-D are representative of at least five mice per group and three independent experiments. **p < 0.01, ****p < 0.0001; n.s., not significant

Human hairy cells arrest at a transitional stage of differentiation between activated B cells and memory B cells, and express dual antigen receptors, IgM/IgD and IgG [6, 45]. To understand molecular mechanisms underlying this phenomenon, we investigated the expression of transcription factors/regulators that are required for germinal center reaction and the following differentiation in HSC^VE^, B^VE^P53^−/−^ and B^VE^PTEN^−/−^ leukemic cells [46]. As shown in Fig. 7A, the expression of most transcription factors/regulators for germinal center reaction and lineage commitment of memory B cell and plasma cell were severely inhibited in HSC^VE^, B^VE^P53^−/−^ and B^VE^PTEN^−/−^ leukemic cells with an extent as B^VE^P53^−/−^ ≈ B^VE^PTEN^−/−^ > HSC^VE^. To validate this RNA-seq data, we carried out quantitative PCR analysis, and found that all leukemic cells down-regulated Batf, Tcf3, Tcf4, Pou2f1 (Oct1), Pou2f2 (Oct2), Mef2b, Mef2c, and Id3 for germinal center reaction [46–53], Bach2 and Hhex for memory B cell differentiation [54, 55], and IRF4 and Prdm1 (Blimp1) for plasma cell differentiation [56, 57], particularly which is to an extremely low level in B^VE^P53^−/−^ and B^VE^PTEN^−/−^ cells (Fig. 7B-D). This data suggests that HSC^VE^, B^VE^P53^−/−^ and B^VE^PTEN^−/−^ leukemic cells lose their ability to mount a germinal center reaction and then differentiate into either memory B cells or plasma cells upon stimulation, though further studies are still needed.

## Discussion

HCL is a chronic B cell lymphoma driven by BRAF(V600E) mutation. However, the origin of hairy cells and concurrent genetic alterations that sustain BRAF(V600E)-driven ontogeny of HCL are not clear. In this study, we have explored the potential concurrent mutations of BRAF(V600E) in HCL by analyzing genomic sequencing data. Using genetically engineered mouse models, we have determined whether these mutations facilitate BRAF(V600E)-driven origination of hairy cells from B lymphocytes. We have demonstrated that BRAF(V600E) mutation together with loss of Trp53 or pTEN in B lymphocytes induces a malignancy with symptoms resembling that of human HCL. Further, we have examined by RNA sequencings the gene expression profiles of leukemic cells from these genetically modified mice and revealed that hairy cells have a unique gene expression signature that could be targeted for disease treatment. Our study has uncovered the B cell origin of hairy cells as well as genetic alterations essential for this event, established an animal model for human disease, and provided molecular basis for developing potential precise targeted therapies in the future, which hence would have important implications for both research and treatment of this disease.

Although HSC^VE^, B^VE^P53^−/−^, and B^VE^PTEN^−/−^ mice develop hematological malignancies, their symptoms are quite different. The disease in HSC^VE^ mice has a much quicker progression, which damages bone marrow, liver and spleen, but also skin, lung and other organs/tissues, in contrast to those in B^VE^P53^−/−^ and B^VE^PTEN^−/−^ mice. The symptoms in HSC^VE^ mice resemble a mixed phenotype of acute leukemia and histiocytosis, but not HCL, although this needs further investigation. This notion is supported by our cellular characterization of HSC^VE^ leukemic cells. Unlike B^VE^P53^−/−^ and B^VE^PTEN^−/−^ leukemic cells, HSC^VE^ leukemic cells rarely form projections on the cell surface and hardly express CD11c, although they have an enlarged cell body and express Annexin A1. In addition, HSC^VE^ leukemic cells have a distinct gene expression signature from those of B^VE^P53^−/−^ and B^VE^PTEN^−/−^ leukemic cells, which exhibits a feature of acute leukemia in our bioinformatic analysis. The symptoms in B^VE^PTEN^−/−^ mice are more aggressive than in B^VE^P53^−/−^ mice, although their overall pathological features are similar. Consistently, B^VE^PTEN^−/−^ leukemic cells have homogeneous rather than heterogeneous expression of CD11c, which likely enhances infiltration. Moreover, B^VE^PTEN^−/−^ leukemic cells also exhibit a different gene expression profile from B^VE^P53^−/−^ leukemic cells, though it is closer to that of B^VE^P53^−/−^ rather than HSC^VE^ leukemic cells. Therefore, we think that leukemia induced by BRAF(V600E) and pTEN^KO^ in B lymphocytes may mimic an aggressive subtype of human HCL that has a high AKT signaling and a poor prognosis. In addition, the long latency of leukemia in B^VE^P53^−/−^ mice and the heterogeneous expression of CD11c on B^VE^P53^−/−^ leukemic cells suggest that extra genetic or epigenetic alterations are still required for the ontogeny of HCL, which will be explored in our future work.

Human hairy cells express multiple Ig isotypes, IgM/IgD and IgG [45], suggesting that they may originate from activated B cells that have been arrested at a stage before differentiating into memory B cell or plasma cells. This notion is strengthened by a recent finding that human hairy cells have a gene expression pattern approximate to that of memory B cells [6]. However, the molecular mechanisms that are responsible for this phenomenon remain largely unknown. In this study, we have addressed this question by profiling the expression of transcription factors/regulators that are essential for germinal center reaction as well as the following lineage commitment in B^VE^P53^−/−^, and B^VE^PTEN^−/−^ leukemic cells. Our data has shown that most of these factors/regulators are extremely down-regulated, suggesting that B^VE^P53^−/−^ and B^VE^PTEN^−/−^ leukemic cells are unable to mount proper germinal center reaction upon stimulation and then to differentiate into memory B cells or plasma cells. However, stronger evidence is still required to completely resolve this issue. Hence, in future studies we will investigate whether B^VE^P53^−/−^ and B^VE^PTEN^−/−^ B-lymphocytes generate memory-like cells that express both IgM/IgD and IgG upon stimulation, and how BRAF(V600E) mutation together with loss of TP53 or PTEN in B-lymphocytes dampens the expression of those transcription factors/regulators for germinal center reaction and memory B cell versus plasma cell differentiation.

Our data has clearly shown how BRAF(V600E) together with loss of tumor suppressor Trp53 or pTEN converts B lymphocytes into malignant cells that are able to induce the symptoms of HCL. However, the exact pathogenesis of HCL as well as its regulatory factors remains ambiguous at present. Our animal models enable us to address these questions by using single cell sequencing methods in the future. The altered cellular programs in hairy cells may produce vulnerabilities that allow us to develop novel therapeutic interventions for HCL, particularly those relapsed or refractory cases.

## Conclusion

Our study indicates that hairy cells originate from B-lymphocytes that harbor BRAF(V600E) mutation and loss of tumor suppressor TP53 or PTEN but not P27. The unique gene expression signature of hairy cells underpins the pathological features of this disease. The animal models constructed in our study will facilitate further understanding of HCL pathogenesis and determining the efficacy of potential therapeutic approaches in the future.

## Materials and methods

### Antibodies and biochemical reagents

Antibodies used in this study for immunoblot include: anti-phospho-ERK1/2 (#4370, Cell Signaling Technologies), anti-ERK1/2 (sc-514302, Santa Cruz), anti-Annexin A1 (#71-3400, Invitrogen), anti-β-actin (A5316, Sigma-Aldrich), and HRP-labeled secondary goat anti-mIgG (#31430, Invitrogen) and anti-rIgG (#31460, Invitrogen). Antibodies used in this study for flow cytometric staining and/or immunohistochemistry staining were: fluorescein-labeled anti-B220 (#103207, clone RA3-6B2, Biolegend), anti-CD19 (#115508, clone 6D5, Biolegend), anti-CD3e (#553061, clone 145-2C11, BD Biosciences), anti-CD11c (#117313, clone N418, Biolegend), anti-CD103 (#121413, clone 2E7, Biolegend), anti-IgD (#17-5993-82, clone 11–26, eBioscience), anti-CD3 (#100240, clone 17A2, Biolegend), anti-B220 (#103228, clone RA3-6B2, Biolegend) antibodies, and Hoechst 33,342 nucleic acid staining reagent (#H3570, Invitrogen) as well as HRP-labeled anti-NFκB (#MAB3026, Merck) antibody.

Genomic DNA extraction kit (#KN-T110005, KANEKA Co., Japan) and Taq DNA polymerase (#M0273, NEB Biolabs) were used for mice genotyping. All other biochemicals were purchased from Sigma-Aldrich.

### Animal studies

All mice strains were obtained from the Jackson laboratory, which include BRAF^CA/+^ (#017837, a Cre-driven BRAF(V600E)-knockin strain) [58], Trp53^flox/flox^ (#008462), pTEN^flox/flox^ (#006440), p27^flox/flox^ (#027328), Mx1-cre (#003556), and cd19^cre/+^ (#006785) mice strains. These strains were bred to generate BRAF^CA/+^Mx1-cre, BRAF^CA/+^ cd19^cre/+^, BRAF^CA/+^Trp53^flox/flox^ cd19^cre/+^, BRAF^CA/+^pTEN^flox/flox^ cd19^cre/+^, BRAF^CA/+^p27^flox/flox^ cd19^cre/+^ mice strains. All mice strains were maintained in the animal facility at the National Cancer Center Singapore (NCCS). To track the disease progression in mice with different genetic modifications, 20 mice per strain were used and monitored up to 40 weeks. To characterize the pathological features of disease in mice, animals at the terminal stage of diseases were euthanized and sacrificed for harvesting of blood, spleen, liver, lung, skin and bone marrow according to standard protocols. All operations were approved by the institutional Animal Care and Use Ethics Committee of NCCS.

### Blood counts measurement and ELISA analysis

Blood samples were collected from anaesthetized mice by tail vein bleeding. Full blood counts were measured using an Abaxis VetScan HM5 hematology analyzer. Serum CD25 was measured by an ELISA kit (#DY2438, R&D system) and following manufacturer’s protocol.

### Lymphocytes isolation, immunostaining, and flow cytometric analysis

Single cell suspensions were prepared respectively from blood, spleens, and bone marrow, and then stained with fluorescein-labeled antibodies using standards procedures. Flow cytometric analyses were performed on a FACSCalibur (BD Biosciences) and analyzed with FlowJo v8.8.6 software (TreeStar, USA).

### Splenic B cell purification, staining and imaging

Splenic B cells were sorted from splenocytes by positive enrichment using anti-CD19 magnetic microbeads (#18954, Stemcell Technologies) as reported before [59]. The purity of B cells as determined by flow cytometric analysis was at all times > 95%. For morphological analysis, purified splenic B cells were seeded on coverslips coated with poly-L-lysine (#P4707, Sigma-Aldrich), fixed with formaldehyde, and stained with Giemsa (#109203, Sigma-Aldrich). All images were acquired using a Nikon Eclipse 90i microscope.

### Immunoblotting

Whole lysate was prepared by lysing cells in RIPA buffer with 1% NP-40, protease inhibitors and phosphatase inhibitors, and the expression of target proteins was detected by immunoblotting as described before [60].

### Histological, immunohistochemistry and immunofluorescent staining

Tissue samples including spleen, liver, bone marrow, skin and lung were fixed for 24 h in 10% formalin, dehydrated, and embedded in paraffin (FFPE) by following standard protocols. A special procedure was applied to decalcify the bones in bone marrow samples before fixation, by immersing the samples in 5% EDTA-Na (pH 7.0) for 7 days. Paraffin blocks were sectioned into 4–5 μm slices, which were then rehydrated and stained with hematoxylin (#HHS16, Sigma-Aldrich) and eosin (#HT110232, Sigma-Aldrich) before imaging.

For immunohistochemistry staining, 5 μm sections of FFPE tissue were mounted onto glass slides, dried overnight at room temperature, and baked at 60 °C for 1 h prior to staining. Then all sections were subjected to rehydration and antigen retrieval using DAKO Target retrieval solution (#S1699, Dako) or citrate (pH 6.0) for 1 h. Next, all samples were blocked with normal goat serum (#5425, Cell Signaling Technologies) or bovine serum albumin for 1 h before incubating with primary antibody labeled with HRP for overnight at 4 °C. The DAB substrate kit, peroxidase (HRP), with Nickel, (3,3’-diaminobenzidine, #SK-4100, Vector Laboratories) was used to produce a brown reaction product in the presence of peroxidase (HRP) enzyme by following the manufacturer’s instructions. Finally, all sections were counterstained with hematoxylin before imaging.

For collagen staining, all sections were rehydrated and stained with Picro Sirius Red (#ab150681, Abcam) according to manufacturer’s protocol before imaging.

For immunofluorescent histology staining, fresh spleens were immersed in cold PBS and transferred to Tissue Tek OCT compound (#4583, Sakura Finetek; #361603E, Lot 03806271, VMR) and cryomold (#4566, Lot 110663, Sakura Tissue Tek). All samples were then immediately frozen in liquid nitrogen and stored in a -80 °C freezer. Frozen specimens were cryosectioned into 10 μm vertical sections with a Leica 3050 S cryostat (Leica Microsystems) maintained at − 20 °C. Following fixation and blocking, all sections were incubated in primary antibody labeled with fluorescein in the dark at 4 °C overnight. Finally, all sections were mounted onto glass slides for fluorescence imaging.

All Images were acquired using a Nikon Eclipse 90i microscope.

### RNA extraction, sequencing and data analysis

RNA samples were prepared from splenic B cells by using an RNA extraction kit (#12183555, Invitrogen) and following the manufacture’s protocol sequenced by using a Gene + Seq-2000 series sequencer and DNBSEQ-T7 sequencing platform (Wuhan Bioacme Biological Technology Co. Ltd and Jiangxi HaploX Genomics Center, HGC). Data was analyzed with Partek® Flow® (Partek®). Briefly, paired-end raw reads were trimmed, removing reads with a Phred score lower than 20 or with length shorter than 25 nucleotides. Trimmed data were aligned to mm10 (*Mus Musculus* genome: mm10_ensembl_release100_v2) with built-in STAR-2.7.3a using default parameters. Filtered gene counts (default) were normalized with CPM (count per million, Add: 1.0E-4). PCA was plotted with six principal components and features contributed equally. Differential analysis was performed with the Partek GSA algorithm. A differentially expressed gene list (filtered feature list) was generated using FDR step up < = 0.05 and Fold change < -2 or > 2. Filtered feature lists from HSC^VE^, B^VE^Trp53^KO^ and B^VE^pTEN^KO^ in comparison with WT counterpart were generated and consolidated before processing with hierarchical clustering to get heatmaps which identify and visualize groups of data. Six groups of genes were of interest for further attention, which reflected the genes that showed differential expression compared to WT counterpart: (1) genes with upregulation in B^VE^Trp53^KO^; (2) genes with upregulation in B^VE^pTEN^KO^; (3) genes with upregulation in HSC^VE^; (4) genes with downregulation in B^VE^Trp53^KO^; (5) genes with downregulation in B^VE^pTEN^KO^; (6) genes showing downregulation in HSC^VE^. For each gene list, pathway analyses were carried out by using the IPA platform (Qiagen) to get the following features: canonical pathways, associated disease and function categories and upstream regulators. To further understand the genes underlying regulation, transcription factors or transcription regulators from these six groups were used for IPA analyses as well. Twenty transcription factors that were reported to involve in B cell germinal center reaction and later differentiation were subjected to hierarchical clustering to highlight the expression patterns in different samples.

### qPCR validation of genes

Top genes in each of the six subgroups were included for validation of RNA-seq data. Genes were excluded when the normalized read in up-regulated sample is lower than 10, and genes showing both high counts and high fold changes were selected. For each sub-group, at least three genes were included for qPCR assays to validate the gene expression indicated by the RNA-seq data. qPCR primers annealing specifically to mouse genes were designed using the online tool Primer3 (https://primer3.ut.ee/). Reverse transcription was carried out with iScript™ cDNA Synthesis Kit (#1708891, Biorad) and cDNAs were amplified using KAPA SYBR® FAST (#KK4617, Merck) on CFX96 or CFX384 real-time PCR detection systems (Biorad). The qPCR data were normalized with Gapdh as an internal control. At least three independent assays (n > = 3) were done for each selected gene.

### Statistical analysis

All statistical analysis was performed using GraphPad InStat (GraphPad Software, USA). Statistical significance was determined by two-tailed Student’s *t*-test in animal studies and error bars represent s.d. to show variance between samples in each group, or by one-sample *t*-test in other experiments and error bars represent s.d. to show variance between independent experiments.

### Supplementary Information

**Additional file 1.**

## Data Availability

All data required for supporting the conclusion in the paper are present in the main text and/or the supplementary materials.

## Abbreviations

HCL: Hairy Cell Leukemia
Trp53: Transformation related protein 53
CDKN1B (P27): Cyclin-Dependent Kinase Inhibitor 1B (p27^Kip1^)
pTEN: Phosphatase and Tensin Homolog
KI: Knockin
KO: Knockout
CCND1: Cyclin D1
CDKN1A (P21): Cyclin-Dependent Kinase Inhibitor 1 A (p21^Cip1^/p21^Waf1^)
KLF2: Kruppel-Like Factor 2
PI3K/AKT: Phosphatidylinositol 3‑kinase/Protein kinase B
FLT3: FMS-like receptor tyrosine kinase-3
bFGF-FGFR1: Basic Fibroblast Growth Factor-Fibroblast Growth Factor Receptor 1
MLL3 (KMT2C): Mixed-Lineage Leukemia Protein 3 (Lysine Methyltransferase 2 C)
KDM6A: Lysine Demethylase 6 A
CREBBP (CBP): Cyclic adenosine monophosphate Response Element Binding Protein Binding Protein (CREB-binding protein)
ARID1A: AT-Rich Interaction Domain 1 A
ARID1B: AT-Rich Interaction Domain 1B
COSMIC: Catalogue of Somatic Mutations in Cancer
ICGC: International Cancer Genome Consortium
MAP2K1 (MEK1): Mitogen-Activated Protein Kinase Kinase 1 (MAPK/ERK Kinase 1)
ERK (MAPK): Extracellular-signal Regulated Kinase (Mitogen-Activated Protein Kinase)
B^VE^P53^−/−^: BRAF^CA/+^Trp53^flox/flox^cd19^cre/+^
B^VE^P27^−/−^: BRAF^CA/+^p27^flox/flox^cd19^cre/+^
B^VE^PTEN^−/−^: BRAF^CA/+^pTEN^flox/flox^cd19^cre/+^
B^VE^: BRAF^CA/+^cd19^cre/+^
HSC^VE^: BRAF^CA/+^Mx1^cre/+^
PCR: Polymerase Chain Reaction
CD25 (IL-2Rα): Cluster of Differentiation 25 (Interleukin-2 Receptor alpha subunit)
IgD: Immunoglobulin D
NFκB: Nuclear factor kappa B
CD11c: Cluster of Differentiation 11c (Integrin alpha X)
CD103: Cluster of Differentiation 103 (Integrin, alpha E (ITGAE))
IPA: Ingenuity Pathway Analysis
VCAM: Vascular Cell Adhesion Molecule
ITGAM: Integrin Subunit Alpha M
Lrp1: LDL Receptor Related Protein 1
Blk: B Lymphocyte Kinase
BLNK: B-cell Linker
Itpr2 (IP3R2): Inositol 1,4,5-Trisphosphate Receptor Type 2
C1qb: Complement Component 1, Q Subcomponent, B Chain
C1qc: Complement Component 1, Q Subcomponent, C Chain
Lamp1: Lysosomal Associated Membrane Protein 1
Trf1: Transferrin Receptor 1
PAK1: P21 Protein (Cdc42/Rac)-Activated Kinase 1
Naaa: N-Acylethanolamine-hydrolyzing Acid Amidase
SLC40A1 (ferroportin): Solute Carrier Family 40 Member 1
ApoE: Apolipoprotein E
C/EBPβ: CCAAT/Enhancer Binding Protein (C/EBP), Beta
MMP9: Matrix Metalloproteinase 9
CTSB: Cathepsin B
Pdia6: Protein Disulfide Isomerase Family A Member 6
PGAM1: Phosphoglycerate Mutase 1
CENP-E: Centromere-Associated Protein E
Xcr1: X-C Motif Chemokine Receptor 1
TLR3: Toll Like Receptor 3
IgM: Immunoglobulin M
IgG: Immunoglobulin G
Batf: Basic Leucine Zipper ATF-Like Transcription Factor
Tcf3: Transcription Factor 3 (E2A immunoglobulin enhancer-binding factors E12/E47)
Tcf4: Transcription Factor 4 (immunoglobulin transcription factor 2)
Pou2f1 (Oct1): POU Domain, Class 2, Transcription Factor 1 (Octamer-Binding Transcription Factor-1)
Pou2f2 (Oct2): POU Domain, Class 2, Transcription Factor 2 (Octamer-Binding Transcription Factor-2)
Mef2b: Myocyte-Specific Enhancer Factor 2B
Mef2c: Myocyte-Specific Enhancer Factor 2 C
Id3: Inhibitor Of DNA Binding 3, Dominant Negative Helix-Loop-Helix Protein
Bach2: BTB Domain And CNC Homolog 2
Hhex: Hematopoietically Expressed Homeobox
IRF4: Interferon Regulatory Factor 4
Prdm1 (Blimp1): PR Domain Zinc Finger Protein 1 (B Lymphocyte-Induced Maturation Protein-1)
CD19: Cluster of Differentiation 19 (B-Lymphocyte Surface Antigen B4)
CD3: Cluster of Differentiation 3
B220 (CD45R/PTPRC): Protein Tyrosine Phosphatase Receptor Type C
HRP: Horseradish Peroxidase
RIPA buffer: Radioimmunoprecipitation Assay buffer
FFPE: Formalin-Fixed Paraffin-Embedded
DAB: 3,3’-Diaminobenzidine
Gapdh: Glyceraldehyde-3-Phosphate Dehydrogenase
