# Supplementary material for: BRAF(V600E) mutation together with loss of Trp53 or pTEN drives the origination of hairy cell leukemia from B-lymphocytes
Source: Mol Cancer. 2023 Aug 5;22:125. doi: 10.1186/s12943-023-01817-8 (PMC10403926; doi:10.1186/s12943-023-01817-8)
Supplement: Supplementary file 1 — Additional file 1. [file 12943_2023_1817_MOESM1_ESM.zip › Updated Supplementary Information.docx]

**Supplementary Information**

**Figure S1.** CD19-Cre-driven knockout of tumor suppressors pTEN, Trp53, and P27 in B-lymphocytes.


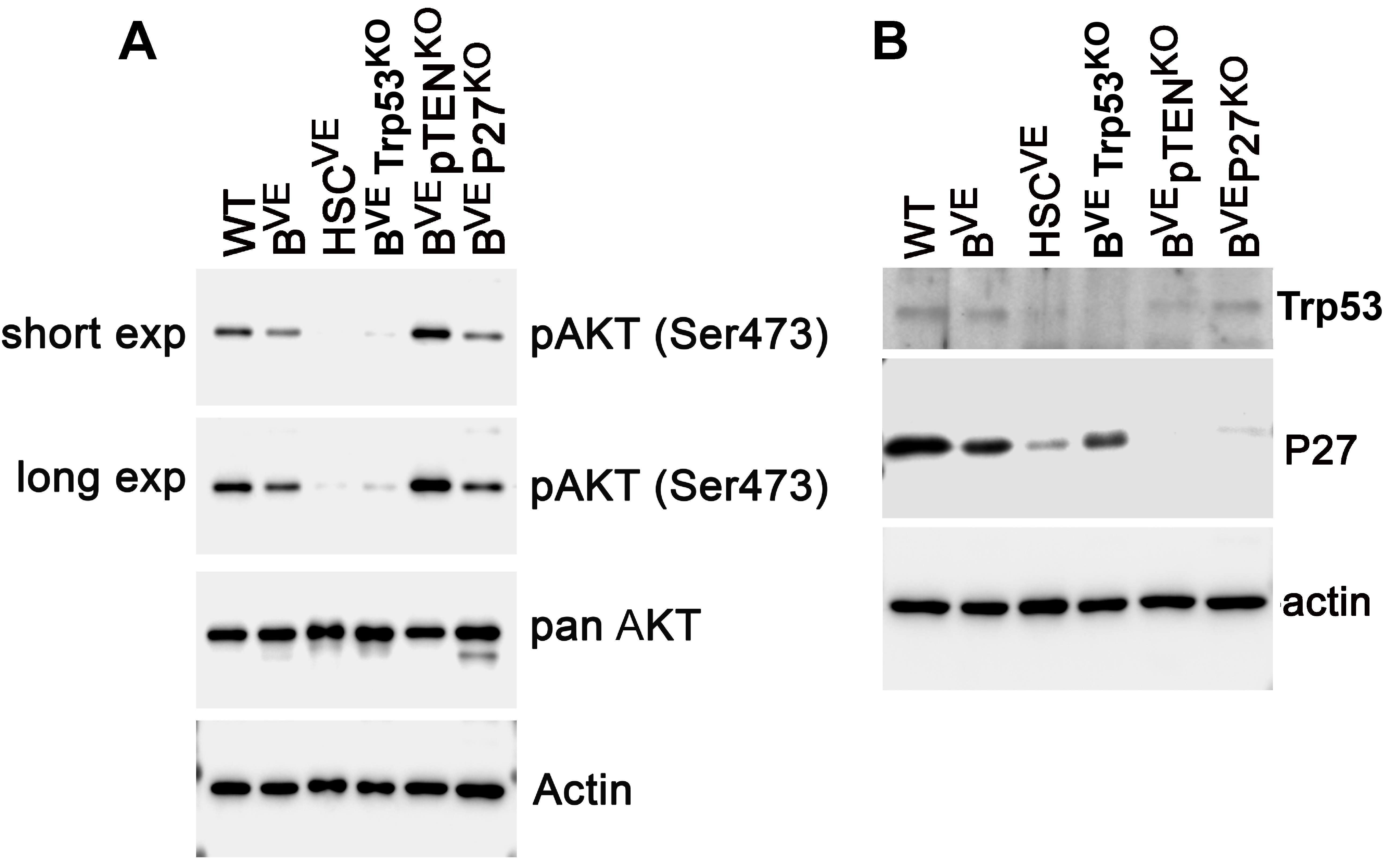


Splenic B cells were isolated from individual mice strains (5-week-old for HSC^VE^ strain, and 10-week-old for all other strains) by using MACS as described in Experimental Materials and Methods, and lysed in RIPA buffer for preparing whole cell lysates. Target proteins in whole cell lysates were detected by SDS-PAGE and immunoblots. A, To confirm the depletion of PTEN, phospho-AKT was examined in whole cell lysates of B-lymphocytes by immunoblot and B^VE^PTEN^-/-^ B-lymphocytes exhibited a much higher level of phospho-AKT than the other samples. B, The depletion of P53 in B^VE^P53^-/-^ B-lymphocytes and P27 in B^VE^P27^-/-^ B-lymphocytes was confirmed by related immunoblots. Surprisingly, the expression of P27 in HSC^VE^ and B^VE^PTEN^-/-^ B-lymphocytes was also inhibited to different extents. All images are representative of at least three independent experiments.

**Figure S2.** HSC^VE^ but not B^VE^P53^-/-^ and B^VE^PTEN^-/-^ mice develop skin inflammation.


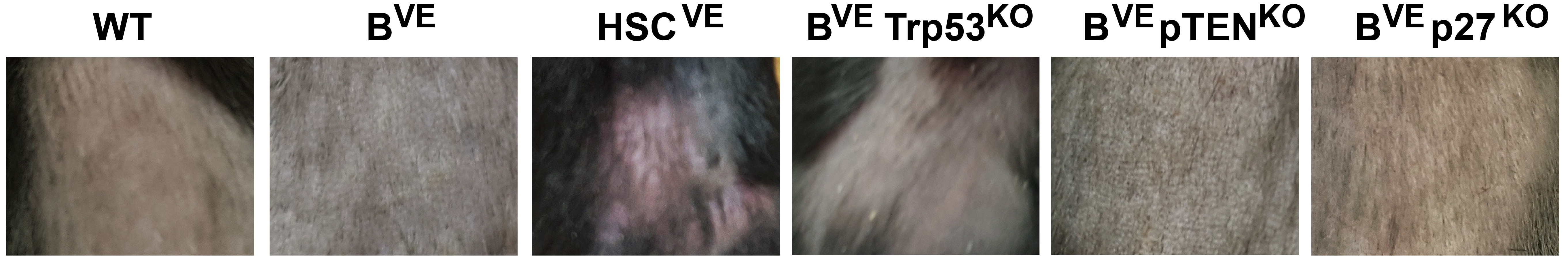


Skin pictures of wild type (25-week), B^VE^ (25-week), HSC^VE^ (7-week), B^VE^P53^-/-^ (25-week), B^VE^PTEN^-/-^ (16-week), and B^VE^P27^-/-^ (25-week) mice were taken by using a digital camera. A skin rash exists in HSC^VE^ mice but not in all other mice. All images are representative of at least three independent experiments.

**Figure S3.** Lymphocyte components were altered significantly in blood, spleen, and bone marrow of HSC^VE^, B^VE^P53^-/-^ and B^VE^PTEN^-/-^ mice at the terminal stage of malignancy.


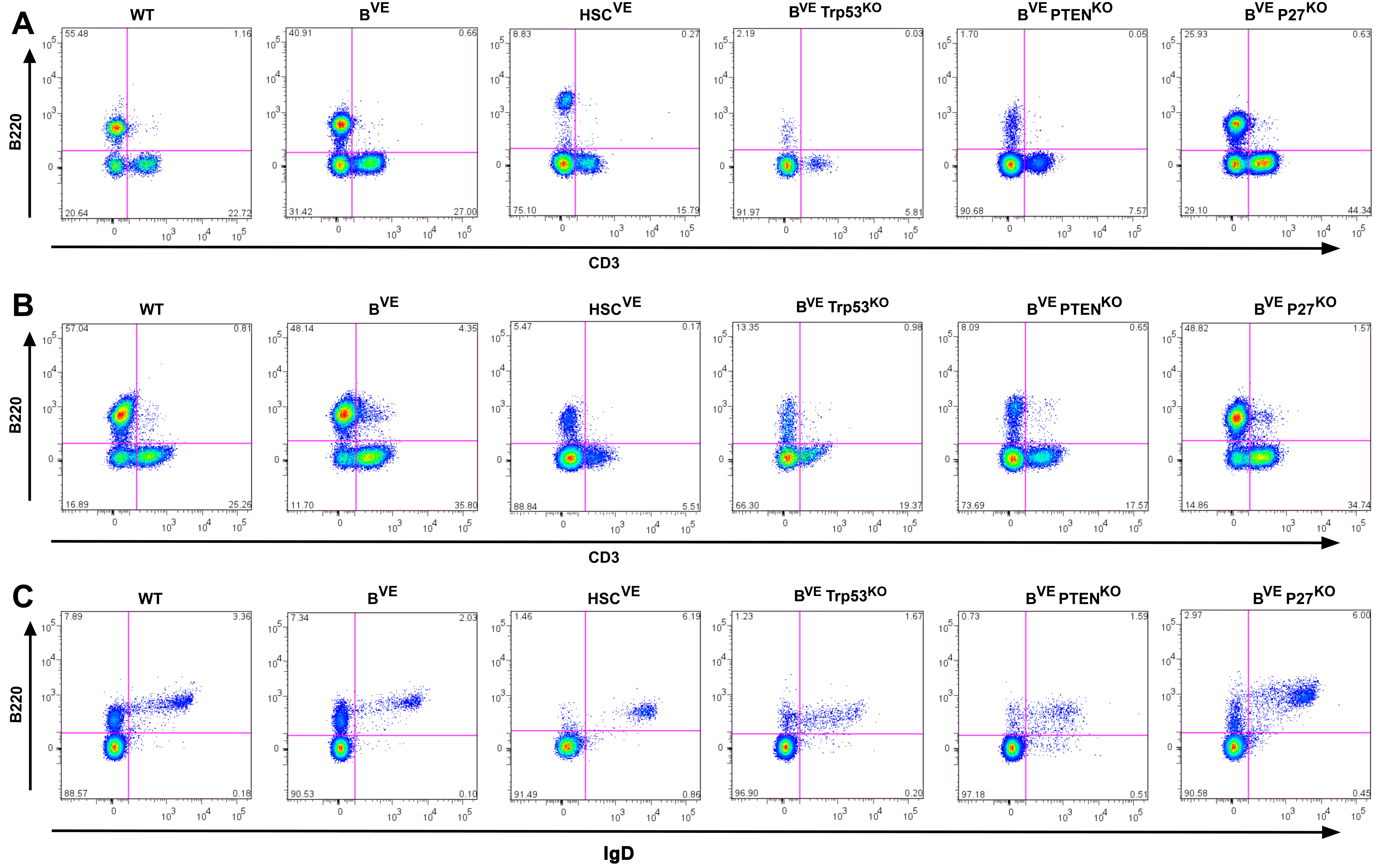


Immune cells were isolated from blood, spleen, and bone marrow as described in Materials and Methods, stained with fluorescein-labeled anti-B220, anti-CD3 or anti-IgD antibodies, and analyzed by flow cytometry. The B cell and T cell counts were extremely reduced, and their ratio was also reversed in spleen and blood of HSC^VE^, B^VE^P53^-/-^ and B^VE^PTEN^-/-^ mice. In bone marrow, the B cell and its progenitors were also extremely reduced in these mice with malignancy, but the ratio of mature B cells (IgD^+^) was significantly increased. All images are representative of at least three independent experiments.

**Figure S4.** The expression of CD11C and CD103 on splenic B cells of HSC^VE^, B^VE^P53^-/-^ and B^VE^PTEN^-/-^ mice at the terminal stage of malignancy.


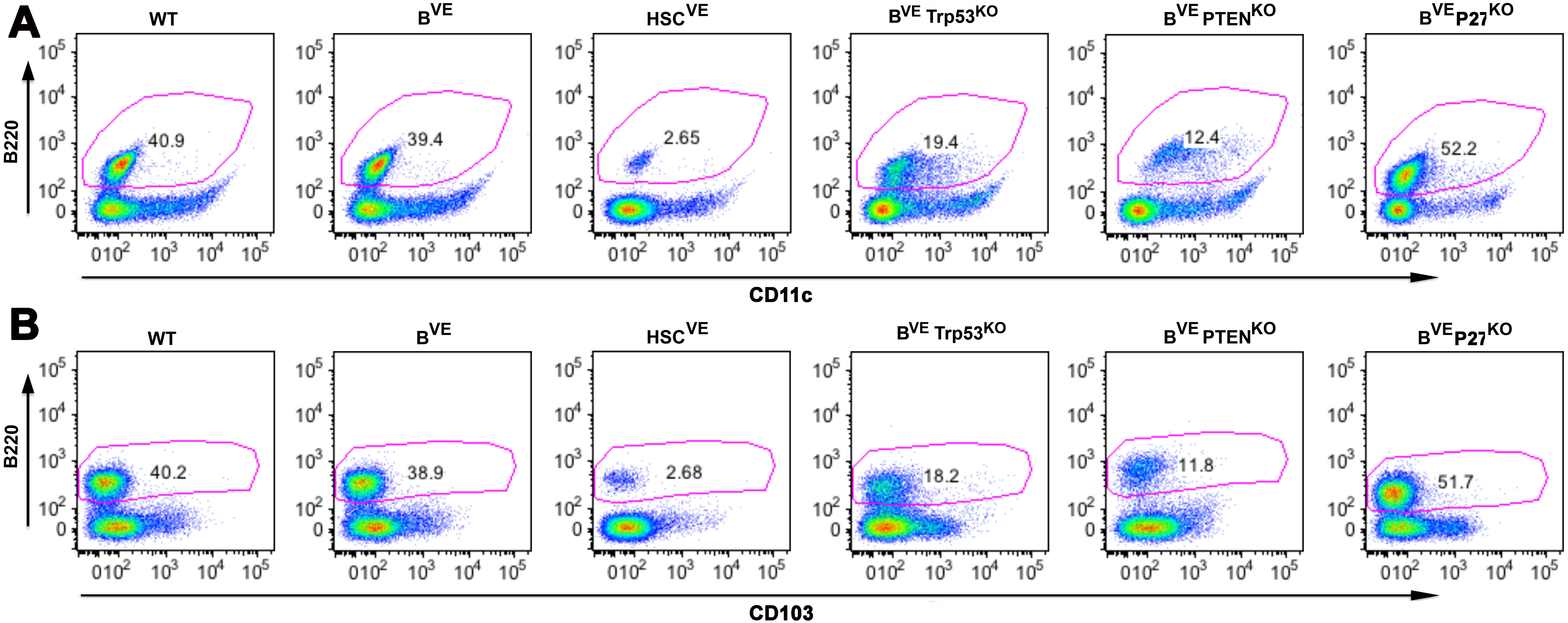


Splenocytes from wild type, B^VE^, HSC^VE^, B^VE^P53^-/-^, B^VE^PTEN^-/-^ and B^VE^P27^-/-^ mice were stained with fluorescein-labeled antibodies and analyzed by flow cytometry. The B220^+^ population was gated to determine the geometric means of CD11c (A) or CD103 (B) in Figure 5B and 5D. All images are representative of at least three independent experiments.

**Figure S5.** The purity of splenic B cells isolated from splenocytes of wild type, B^VE^, HSC^VE^, B^VE^P53^-/-^, B^VE^PTEN^-/-^, and B^VE^P27^-/-^ mice using MACS.


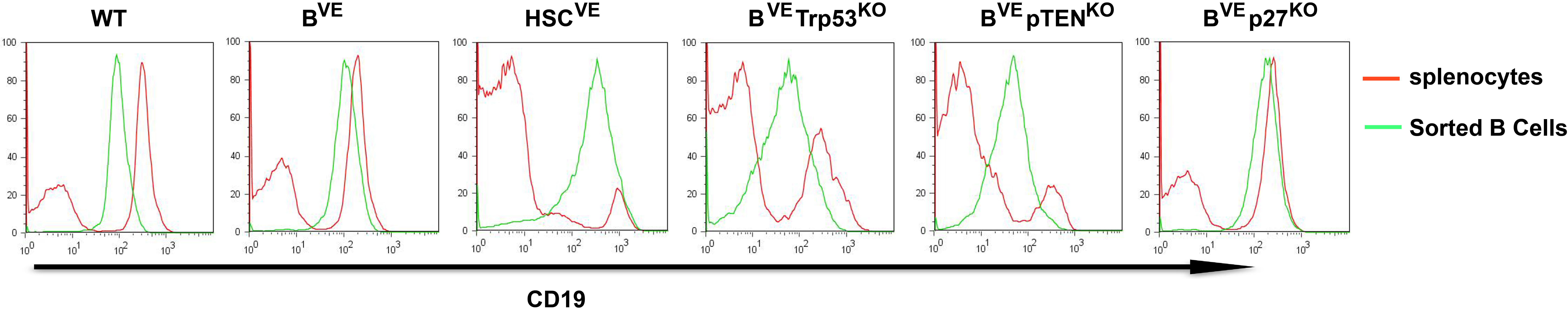


The purity of splenic B cells was measured by anti-CD19 staining and flow cytometry. Splenic B cells were isolated from HSC^VE^, B^VE^P53^-/-^ and B^VE^PTEN^-/-^ mice at the terminal stage of malignancy, or all other mice strains of 25-week-old. All images are representative of at least three independent experiments.

**Table S1. Genetic mutations in human HCL samples.**

| Gene | Samples with mutations | Samples sequenced | Frequency |
| --- | --- | --- | --- |
| BRAF | 843 | 997 | 85% |
| MAP2K1 | 28 | 268 | 11% |
| TP53 | 27 | 185 | 15% |
| CDKN1B | 17 | 174 | 10% |
| KMT2C | 14 | 89 | 16% |
| ARID1A | 11 | 123 | 9% |
| CREBBP | 8 | 123 | 7% |
| KDM6A | 8 | 120 | 7% |
| U2AF1 | 5 | 165 | 3% |
| ARID1B | 5 | 120 | 4% |
| NOTCH1 | 4 | 124 | 3% |
| CEBPA | 4 | 92 | 4% |
| RUNX1 | 3 | 93 | 3% |
| AR | 3 | 89 | 3% |
| GRIN2A | 3 | 89 | 3% |
| FAT4 | 3 | 28 | 11% |
| ZBTB16 | 3 | 25 | 12% |
| CO2A1 | 3 | 25 | 12% |
| NOTCH2 | 2 | 138 | 1% |
| EZH2 | 2 | 124 | 2% |

Data was collected and analyzed using the Cancer Browser from the COSMIC database, and the analyzed link of hairy cell leukemia is:

https://cancer.sanger.ac.uk/cosmic/browse/tissue?wgs=off&sn=haematopoietic_and_lymphoid_tissue&ss=all&hn=all&sh=hairy_cell_leukaemia&in=t&src=tissue&all_data=n.

The hairy cell leukemia cases from the ICGC (International Cancer Genome Consortium) database, and the cBioportal for Cancer Genomics database as well as those in the literatures were also included here if they had been sequenced for mutated genes.
